# European Union citizens’ views on development assistance for developing countries, during the recent migrant crisis in Europe

**DOI:** 10.1186/s12992-018-0378-1

**Published:** 2018-06-28

**Authors:** Alexander Huepers, Henock B. Taddese, Filippos T. Filippidis

**Affiliations:** Department of Primary Care and Public Health, School of Public Health, Imperial College London, 310 Reynolds Building, St. Dunstan’s Road, London, W6 8RP UK

**Keywords:** Cross-sectional survey, Development aid, Public opinion, Migration

## Abstract

**Background:**

Development assistance from governments of high income countries represents the vast majority of international funding for global health. Recent stagnation of this important source of funding may affect attainment of major global health goals. The financial crisis is widely accredited as denting governments’ outlay for development aid, as well as citizen’s support for aid. Europe has also recently experienced record levels of migration; the so called ‘European migration crisis’. This study aims to analyse trends in public attitudes towards development aid in European Union (EU) countries, in the context of the European migrant crisis.

**Methods:**

Eurobarometer survey data from 2011 (prior to the migrant crisis) and 2015 (at the peak of the crisis) was analysed for 27 EU countries. The outcome variables related to people’s levels of support to three statements around the importance of supporting people in developing countries, increasing countries’ commitments to aid and willingness to pay extra for products from developing countries. EU Member States were categorised as ‘arrival’ or ‘destination’ countries in view of migration routes and numbers of asylum applications per 100,000 population, respectively. Multiple linear regression analysis was performed, adjusting for countries’ economic status (gross domestic product per capita).

**Results:**

In general, support for development aid has increased from 2011 to 2015, but was largely unaffected by migration status when applying the regression model. In 2015, the belief that development assistance is ‘very important’ was significantly higher in countries where migrants first arrived compared to other EU Member States, with a trend towards this association also apparent in 2011.

**Conclusions:**

The positive trends in public support for development aid are encouraging in an age where economic hardships at home, as well as the tone of national political discourses and rising right wing populism appear to suggest otherwise.

**Electronic supplementary material:**

The online version of this article (10.1186/s12992-018-0378-1) contains supplementary material, which is available to authorized users.

## Background

Despite the rising influence of civil societies and private corporations, governments of high income countries still provide the vast majority of internationally sourced funding for global health [1]. While this support from governments, known as Official Development Assistance for Health (ODA-H), had risen sharply in the first decade of this century, it has plateaued since 2010 [1]. Furthermore, ODA-H commitments from European Union Development Assistance Committee (DAC) members of the Organisation for Economic Co-Operation and Development (OECD) were substantially lower in 2014 and 2015 compared to previous years [2]. Still, ODA-H remains a major determinant of global health outcomes, as it represents a relatively stable source of funding for major global health programmes. Disease control programmes, such as malaria control and elimination interventions, and wider health system strengthening initiatives in developing countries, may only be adequately supported if the rate of increase returns to levels observed between 2000 and 2010 [3, 4].

The European Union (EU) and its Member States are the leading donors of development aid worldwide. They provided over half the total ODA of OECD DAC members in 2015 [5]. Health is just one sub-sector of ODA; still other programmes targeting a range of fields including education, energy, agriculture and environment can have a major impact on health [6].

ODA is a long way off from the vision set for OECD DAC members, which specified a commitment of 0.7% of their respective gross national incomes (GNI). This target, set in the 1970s, had been met by just six OECD countries in 2015: Denmark, Luxembourg, Sweden, the Netherlands, Norway and the United Kingdom [5]. Several socio-political and economic factors are said to have further slowed progress towards the 0.7% target in many OECD countries [7].

Following the global financial crisis of 2008, most EU donor countries have shown reluctance to increase their development budgets citing prevailing economic difficulties at home [8]. The amount of money spent on foreign aid has also become a major feature of the political discourse in European countries, as most recently evident in the 2017 general election campaigns in the UK [9]. Development programmes have been drawn into national debates over the merits of development aid or the perceived profligacy of the aid budget [10]. The recent rise of right-wing and nationalist populism also casts shadow over the resolve of parliaments across the EU to uphold ODA commitments [11]. This increased politicisation of foreign aid in turn renders the exercise of gauging and describing public opinion on the matter critically important [12, 13].

Generally, public support for various aspects of European development aid has been found to be consistently high over the past decades. A survey of 24,999 people in 2004 showed that 91% of European citizens believed helping people in developing countries to be important [14]. This figure was 88% in 2009, and 89% in 2010, thereby showing no sign of denting in the face of the financial crisis [15, 16]. On the contrary, the proportion of EU citizens who show strong support for this issue by indicating their belief that development aid is ‘very important’ fell from 53% in 2004 to 39% in 2009 (measured on a scale including the following options: ‘very important’, ‘fairly important’, ‘not very important’, ‘not at all important’). Hence, while general support has remained stable over the years, strong opinions on the matter (‘very important’) seem to be more sensitive to changing times. Despite this observation, analysis in most of the extant literature tends to categorise the ‘very important’ and ‘fairly important’ measures together [17, 18], thereby potentially obscuring shifting trends in public perspectives towards development aid.

Besides economic hardships caused by the financial crisis, shifts in public perception may be associated with the migration crisis that peaked in recent years, especially in Europe. Surveys deployed as part of the new 2016 European Consensus on Development highlighted that EU citizens identified the topic of migration as especially important to address [19]. Worldwide, at the end of 2015, an unprecedented 65.3 million people were forcibly displaced from their homes due to violence, political unrest or violations of human rights [20]. The number of illegal border crossings into the EU detected by the European Border and Coast Guard Agency (Frontex) in 2015 was more than six-times greater than the already record-high numbers of 2014. The majority of migrants were displaced individuals from Syria, Afghanistan and Iraq, as well as African countries such as Eritrea and Somalia [21]. Migrants mainly arrive in south-eastern EU countries, often lacking sufficient food and water and requiring medical assistance [22]. Many continue their journey, aiming to seek asylum and settle in Western and Central European nations [21]. This unprecedented flow of migrants began to feature extensively in the news in the summer and autumn of 2015, and was labelled the ‘European migrant crisis’ [23].

This study aims to describe recent opinions across the EU on development aid, and analyse the factors that may be responsible for differences across time and between countries, comparing the peak year of the migration crisis (2015) with a preceding year (2011). It is hypothesised that the migrant crisis may have affected EU citizens’ attitudes towards development aid, particularly in countries where migrants arrive or attempt to settle down permanently. Apart from official European Commission reports, there is lack of research addressing recent trends in EU citizens’ opinions on development aid and any associated factors. This study may fill critical gaps in that regard.

## Methods

### Data source

Two primary datasets, obtained by TNS Opinion (Brussels), were used in the analysis: Special Eurobarometer wave 76.1, conducted in September 2011 (*n* = 26,856), and wave 84.4 from December 2015 (*n* = 27,672) [24, 25]. The Eurobarometer is a series of annual surveys gauging public opinion on a number of different socio-political issues, including development aid. A systematic sampling process, based on administrative regional units as defined by the European Commission’s Nomenclature of Territorial Units for Statistics Level 2 (NUTS 2), was used in each of the 27 EU Member States (EU27). This process selected participants aged 15 or above into a representative sample size as per the countries’ population size and regional population density. Participants were then interviewed face-to-face at home in their respective national language. Post-stratification and population weights were used to ensure representativeness of the samples.

Other data were drawn from Eurostat, the statistical office of the EU. For each EU member state, the following figures were obtained for 2011 and 2015: total population on 1 January, number of asylum applicants, and gross domestic product (GDP) per capita in Euros (EUR) [26–28]. All datasets were de-identified and publicly available; hence no ethical approval was required.

### Measures

The Eurobarometer surveys contained three questions, identical in 2011 and 2015, which were of interest to this study. Participants were asked “In your opinion, is it very important, fairly important, not very important or not at all important to help people in developing countries?”. Answer options included ‘very important’, ‘fairly important’, ‘not very important’, ‘not at all important’ and ‘don’t know’. In the present study, responses were grouped to create a binary variable for strong support (‘very important’ vs. other options) and a variable for general support (‘very important’ & ‘fairly important’ vs. other options).

The surveys also included the question “The EU (the European Commission and Member States) has promised to increase the level of its aid towards developing countries. Given the current economic situation, which of the following statements best describes your opinion?”. Response options were ‘we should increase aid to developing countries beyond what is already promised’, ‘we should keep our promise to increase aid to developing countries’, ‘we should not increase aid to developing countries even though it has been promised’, ‘we should reduce aid to developing countries as we can no longer afford it’ and ‘don’t know’. This was also recoded into a binary variable for strong support (‘increase beyond promise’ vs. other options) and a variable for general support (‘increase beyond promise’ & ‘keep promise’ vs. other options).

Participants were also asked “Would you be prepared to pay more for groceries or products from developing countries to support people living in these countries (for instance for fair trade products)?”. Possible answers included ‘no, you are not ready to pay more’, ‘yes, you would be ready to pay up to 5% more’, ‘yes, you would be ready to pay 6 to 10% more’, ‘yes, you would be ready to pay more than 10% more’ and ‘don’t know’. For this question, a binary variable to indicate preparedness to pay any amount for products from developing countries was created (‘ready to pay more for products’ vs. ‘not ready’).

**Fig. 1:**
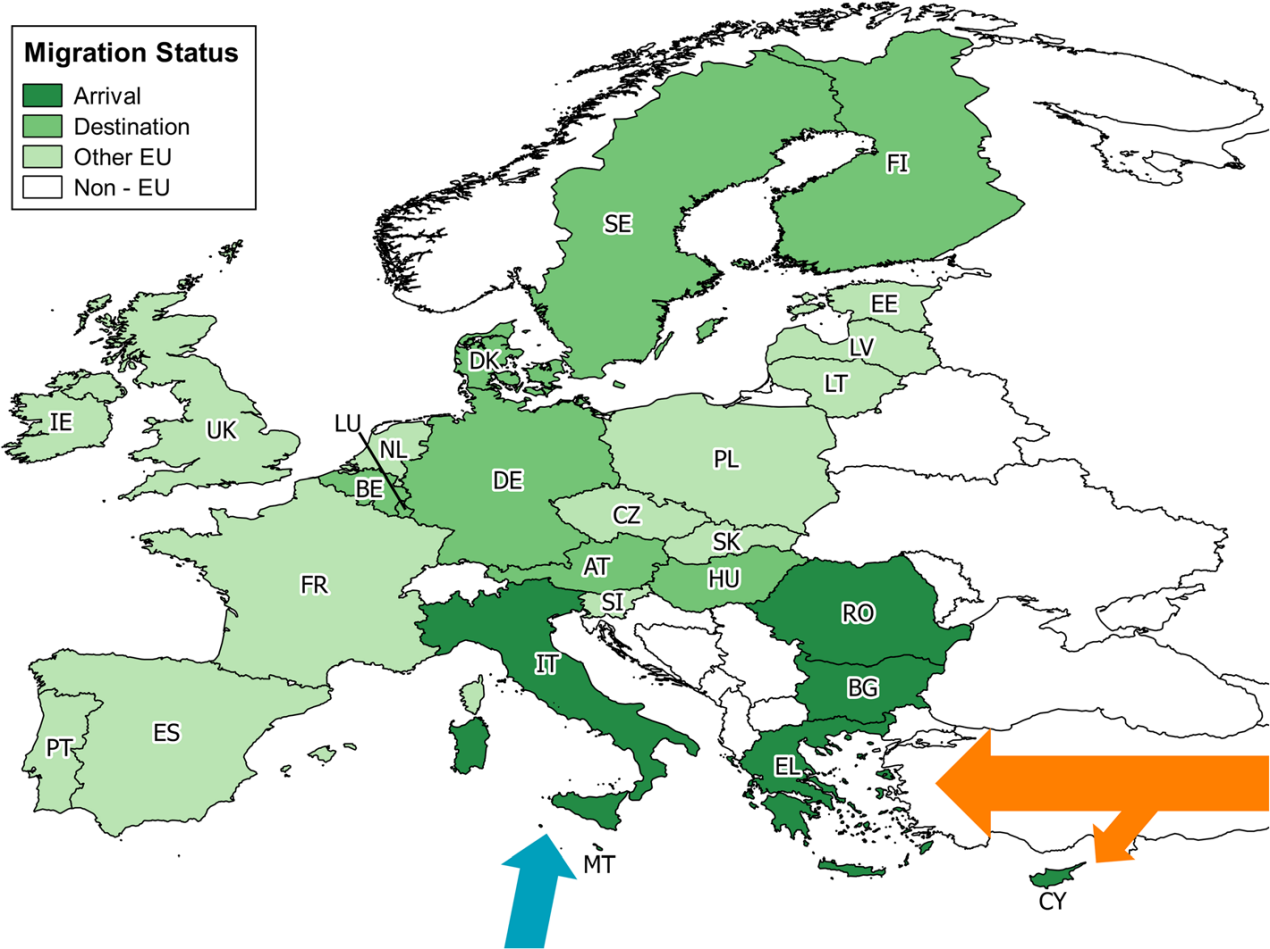
The 27 EU Member States and their designated Migration Status. The arrows illustrate the two major paths of migration into the EU by which ‘arrival’ status was determined: The Eastern Mediterranean route (orange) and Central Mediterranean route (blue). The map’s base-layer was taken from the European Commission’s reference data for countries [47]

These binary variables will henceforth be referred to as ‘Development Views’ collectively. Changes between 2011 and 2015 were calculated relative to 2011 values. The number of asylum applicants was divided by country population to obtain asylum applicants per 100,000 population. Each country was then classed as ‘arrival’, ‘destination’, or ‘other EU’ country. Arrival countries were defined as any EU Member State migrants can first enter via the Eastern Mediterranean or Central Mediterranean routes, as classified by Frontex [21]. These are Bulgaria, Cyprus, Greece, Italy, Malta and Romania. A country was classified as Destination if it was amongst the top ten for asylum applications per 100,000 population in 2015 [27]. These were Hungary, Sweden, Austria, Finland, Germany, Luxembourg, Belgium and Denmark. Bulgaria and Malta, which qualified for both arrival and destination status, were classified as arrival only, due to the relative importance of their status as ports of entry into the EU and their low position amongst the top ten countries for asylum applications (tenth and seventh respectively). All countries not given a status were labelled as ‘other EU’. The migration status was added to the dataset as a categorical variable (‘other EU’; ‘arrival’; ‘destination’). Figure 1 shows the migration status of the EU27 Member States.

### Statistical analysis

Weighted percentages of responses for each of the assessed Eurobarometer questions were estimated at the national level using the weights provided in the official dataset to account for the complex sampling design. An ecological analysis with member state as the unit of analysis was conducted. All data and variables were collated, and multiple linear regression analysis was performed. For both 2011 and 2015, the Development Views were chosen as dependent variables (‘very important’, ‘increase beyond promise’, ‘pay more’). Independent variables included migration status and GDP per capita (per thousand EUR) of the relevant year. Linear regression models with percentage change of these Development Views from 2011 to 2015 as the dependent variable were also run; migration status and percentage change in GDP per capita were used as independent variables. All independent variables were assessed for statistical significance at *p* < 0.05.

All statistical analyses were done using STATA Statistical Software, Version 13.1 [29]. Maps were created with QGIS Geographic Information System, Version 2.18.6. [30]. Descriptive results are presented as weighted percentages. Regression results are shown as beta-coefficients with 95% confidence intervals (CI).

## Results

In the EU27, support for development aid being very important increased by 10.5%, from 35.9% in 2011 to 39.7% in 2015. The highest proportion of EU citizens who believe that it is very important to help people in developing countries in 2011 was found in Cyprus (74.0%), with a low of 19.7% in Estonia. Sweden (70.4%) and Latvia (15.7%) were highest and lowest in 2015. The greatest relative increases from 2011 to 2015 were found in Romania, Slovenia and Ireland. Citizens of Lithuania, Latvia, Slovakia and Poland were least supportive in 2015 compared to 2011. Table 1 illustrates percentages, and relative change from 2011 to 2015, for all EU countries.

**Table 1:** Percentage of each EU country’s population supporting the Development View ‘very important’, 2011 and 2015

Country	Opinion ‘very important’, 2011 (%, 95 CI)	Opinion ‘very important’, 2015 (%, 95 CI)	Change in ‘very important’, 2011–2015 (%)
Austria (AT)	34.7 (31.7–37.7)	38.2 (34.9–41.6)	10.1
Belgium (BE)	36.2 (33.2–39.3)	39.7 (36.6–43.0)	9.8
Bulgaria (BG)	23.3 (20.7–26.1)	25.2 (22.6–28.0)	8.0
Cyprus (CY)	74.0 (70.0–77.6)	66.7 (62.1–71.1)	−9.8
Czech Republic (CZ)	24.1 (21.6–26.8)	25.2 (22.5–28.1)	4.5
Denmark (DK)	51.4 (48.1–54.8)	46.5 (43.1–50.0)	−9.5
Estonia (EE)	19.7 (17.3–22.5)	17.3 (14.9–20.0)	−12.4
Finland (FI)	38.7 (35.4–42.1)	41.2 (37.8–44.6)	6.3
France (FR)	35.3 (32.4–38.4)	35.9 (32.9–38.9)	1.5
Germany (DE)	53.1 (50.2–56.0)	52.7 (49.6–55.7)	−0.8
Greece (EL)	30.8 (27.9–33.8)	40.3 (37.0–43.6)	30.8
Hungary (HU)	19.9 (17.4–22.6)	21.4 (18.9–24.3)	7.9
Ireland (IE)	40.8 (37.7–43.9)	58.2 (55.0–61.4)	42.9
Italy (IT)	24.8 (22.2–27.6)	29.5 (26.5–32.8)	19.0
Latvia (LV)	22.7 (20.2–25.5)	15.7 (13.4–18.3)	− 30.8
Lithuania (LT)	28.9 (26.2–31.8)	16.8 (14.1–19.9)	−41.8
Luxembourg (LU)	59.4 (54.8–63.8)	54.5 (49.4–59.5)	−8.2
Malta (MT)	46.3 (41.5–51.3)	62.0 (57.1–66.7)	33.8
Netherlands (NL)	34.2 (30.6–38.0)	45.9 (42.6–49.3)	34.2
Poland (PL)	31.7 (28.8–34.8)	23.8 (21.2–26.7)	−24.7
Portugal (PT)	25.8 (23.2–28.5)	27.3 (24.6–30.2)	5.9
Romania (RO)	28.3 (25.6–31.2)	45.9 (42.7–49.2)	62.3
Slovakia (SK)	29.1 (26.1–32.3)	21.4 (18.9–24.2)	−26.3
Slovenia (SI)	20.9 (18.4–23.5)	33.4 (30.3–36.6)	60.1
Spain (ES)	36.4 (33.4–39.5)	46.4 (42.9–49.9)	27.5
Sweden (SE)	69.0 (65.7–72.2)	70.4 (66.2–74.3)	2.0
United Kingdom (UK)	34.9 (32.0–37.9)	44.2 (41.1–47.4)	26.6
**EU27**	**35.9 (35.0–36.8)**	**39.7 (38.7–40.7)**	**10.5**

This table illustrates the percentage of each country’s population of opinion that helping people in developing countries is ‘very important’, 2011 and 2015

Across the EU27 as a whole, a 32.6% increase in the percentage of people in favour of seeing EU development aid increased beyond what was promised was observed (12.0% in 2011, 15.9% in 2015). In 2011, levels of support ranged from 24.5% in Austria to 3.2% in Bulgaria. Respondents in Bulgaria remained least supportive in 2015 (2.6%), while neighbours Romania were most supportive (29.1%) – a relative increase of 157.8% compared to 2011. Other countries with the greatest increases included Ireland, Cyprus, Slovenia and Malta. Support of increase beyond the EU’s promise declined the most in Poland, from 14.0% in 2011 to 7.0% in 2015. Table 2 shows percentages for 2011 and 2015, as well as the relative change, for all 27 EU Member States. For levels of general support, see Additional file 1: Table S1.

**Table 2:** Percentage of each country’s population supporting the Development View ‘increase beyond promise’, 2011 and 2015

Country	Opinion ‘increase beyond promise’, 2011 (%, 95 CI)	Opinion ‘increase beyond promise’, 2015 (%, 95 CI)	Change in ‘increase beyond promise’, 2011–2015 (%)
Austria (AT)	24.5 (21.9–27.3)	20.0 (17.3–22.9)	−18.5
Belgium (BE)	12.2 (10.3–14.4)	12.7 (10.6–15.1)	4.6
Bulgaria (BG)	3.2 (2.2–4.5)	2.6 (1.8–3.9)	−17.1
Cyprus (CY)	7.3 (5.3–9.9)	22.7 (19.0–26.9)	213.4
Czech Republic (CZ)	9.1 (7.5–11.0)	7.3 (5.8–9.2)	−19.7
Denmark (DK)	14.1 (12.0–16.6)	14.2 (12.0–16.7)	0.8
Estonia (EE)	5.4 (4.1–7.1)	5.8 (4.4–7.7)	7.6
Finland (FI)	5.4 (4.1–7.2)	5.6 (4.2–7.4)	2.3
France (FR)	12.4 (10.5–14.7)	19.9 (17.5–22.5)	59.8
Germany (DE)	11.8 (10.1–13.7)	16.5 (14.3–18.9)	40.0
Greece (EL)	12.8 (10.8–15.0)	11.3 (9.4–13.6)	− 11.3
Hungary (HU)	6.2 (4.9–8.0)	11.7 (9.7–14.0)	86.6
Ireland (IE)	5.8 (4.4–7.5)	18.9 (16.5–21.6)	226.4
Italy (IT)	16.8 (14.5–19.2)	16.2 (13.9–18.9)	−3.1
Latvia (LV)	9.7 (8.0–11.7)	8.8 (7.0–10.9)	−9.3
Lithuania (LT)	5.0 (3.9–6.5)	4.8 (3.4–6.7)	−3.9
Luxembourg (LU)	12.9 (10.2–16.3)	13.1 (10.2–16.8)	1.5
Malta (MT)	7.2 (5.1–10.1)	15.6 (12.4–19.4)	117.1
Netherlands (NL)	8.3 (6.2–10.9)	10.7 (8.8–12.9)	29.0
Poland (PL)	14.0 (11.9–16.4)	7.0 (5.5–8.8)	−50.3
Portugal (PT)	8.9 (7.3–10.8)	15.9 (13.7–18.4)	78.8
Romania (RO)	11.3 (9.5–13.4)	29.1 (26.2–32.2)	157.8
Slovakia (SK)	7.8 (6.1–9.9)	6.7 (5.2–8.6)	−13.8
Slovenia (SI)	8.2 (6.6–10.0)	19.3 (16.8–22.0)	135.6
Spain (ES)	13.7 (11.7–16.0)	25.6 (22.7–28.8)	87.0
Sweden (SE)	12.7 (10.5–15.2)	12.9 (10.2–16.0)	1.5
United Kingdom (UK)	8.9 (7.2–10.9)	14.2 (12.1–16.5)	59.1
**EU27**	**12.0 (11.4–12.7)**	**15.9 (15.2–16.7)**	**32.6**

This table illustrates the percentage of each country’s population of opinion that development aid should be ‘increased beyond promise’ of the EU, 2011 and 2015

**Fig. 2:**
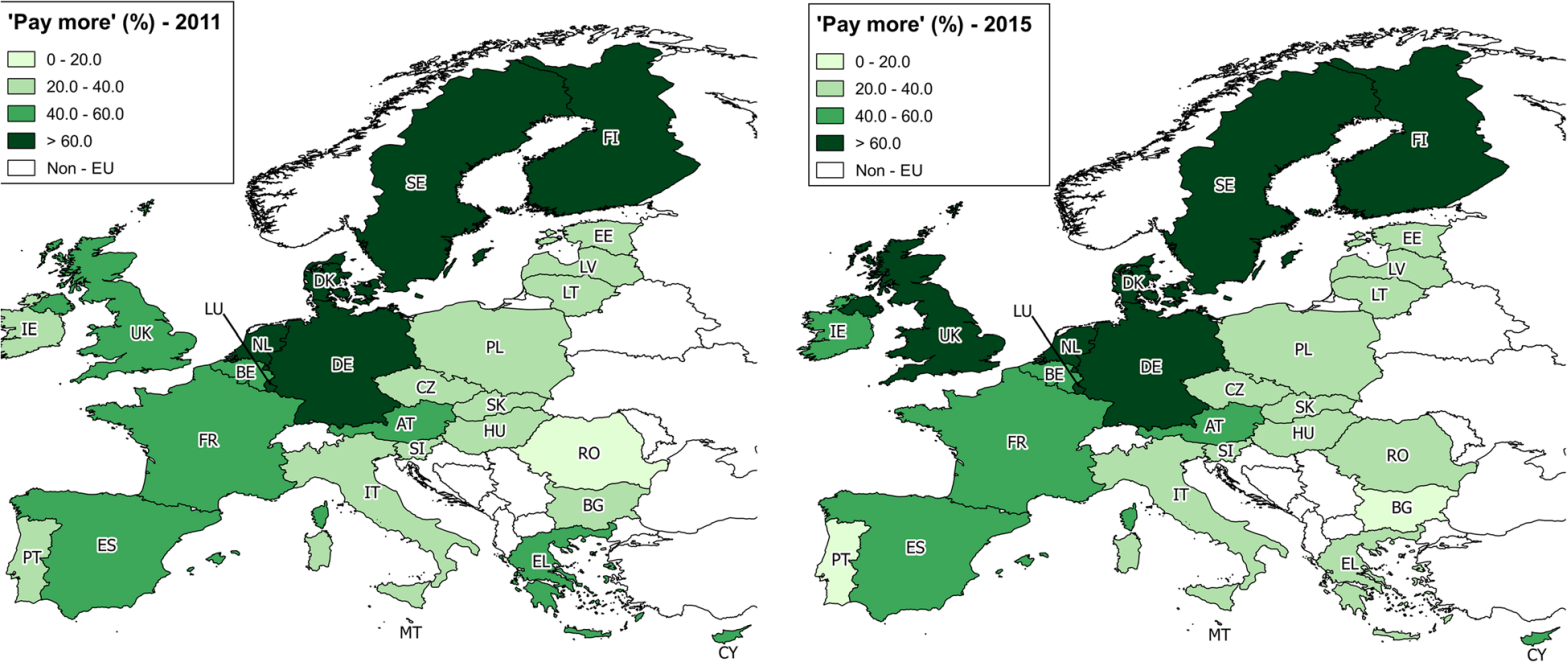
Percentage of citizens prepared to pay more for products from developing countries, 2011 and 2015. The map’s base-layer was taken from the European Commission’s reference data for countries [47]

The EU27 saw a 4.7% relative increase in preparedness of its citizens to pay more for products from developing countries to support the people living there, from 47.3% in 2011 to 49.5% in 2015. In 2011, the greatest support was found in the Netherlands (79.4%), and the lowest in Romania (19.4%). In 2015, percentages ranged from 80.3% in Sweden to 15.5% in Bulgaria. Romania represented the greatest increase (38.0%), while the biggest declines were seen in Bulgaria (30.3%), Lithuania, Poland and Greece (25.8%). Figure 2 illustrates levels of preparedness to pay more for products from developing countries in 2011 and 2015 (also see Additional file 1: Table S2).

In 2011, the percentage of citizens who believed helping people in developing countries to be very important was on average 10.71 percentage points (pp) (− 0.68 to 22.09) higher in arrival countries compared to other EU countries, representing a borderline statistically significant association. A 1000 EUR rise in GDP per capita was associated with an increase of 0.56 pp. (0.22 to 0.92) in support. In 2015, the association between arrival countries and the ‘very important’ opinion was statistically significant, with an average of 17.22 pp. (5.39 to 29.05) higher support. A rise in GDP per capita was again significantly associated with greater support (β = 0.66, 0.32 to 1.00). No significant associations were found between destination status and the three Development Views in any of the models. Table 3 shows beta-coefficients and *p* values, with each of the Development Views as dependent variables. For sensitivity analyses with alternative classification of Development Views, see Additional file 1: Table S3.

**Table 3:** Association of Development Views with migration status and GDP per capita, 2011 and 2015

	Very important 2011		Very important 2015		Increase beyond promise 2011		Increase beyond promise 2015		Pay more 2011		Pay more 2015	
	β (95% CI)	p	β (95% CI)	p	β (95% CI)	p	β (95% CI)	p	β (95% CI)	p	β (95% CI)	p
Migration status												
Other EU country	(referent)											
Arrival country	10.71 (−0.68 to 22.09)	0.064	17.22 (5.39 to 29.05)	0.006	1.06 (−3.39 to 5.50)	0.628	4.31 (−2.55 to 11.18)	0.206	−1.91 (−12.14 to 8.33)	0.704	0.63 (−11.13 to 12.38)	0.913
Destination country	5.04 (−7.20 to 17.28)	0.403	2.19 (−10.04 to 14.42)	0.714	1.99 (−2.79 to 6.77)	0.397	−1.48 (−8.57 to 5.62)	0.671	5.21 (−5.80 to 16.22)	0.338	8.02 (−4.13 to 20.17)	0.185
GDP/ capita (per 1000 EUR)	0.57 (0.22 to 0.92)	0.003	0.66 (0.32 to 1.00)	0.001	0.08 (−0.06 to 0.22)	0.252	0.12 (−0.08 to 0.32)	0.226	0.81 (0.49 to 1.13)	< 0.001	0.90 (0.56 to 1.24)	< 0.001

Shown for each covariate are regression coefficient β, 95% CI for β, and *p* value of statistical significance. β coefficients are adjusted for all variables shown in the table

Arrival status and GDP per capita were not significantly associated with the proportion of respondents supporting the EU to increase development aid beyond what is promised in 2011 (arrival: β = 1.06, − 3.39 to 5.50; GDP per capita: β = 0.08, − 0.06 to 0.22). Similarly, in 2015, the level of support for increasing aid beyond what was promised was not found to be statistically significant with migration status (β = 4.31, − 2.55 to 11.18) and GDP per capita (β = 0.12, − 0.08 to 0.32).

Compared to other EU countries, citizens of arrival countries were not prepared to pay significantly more for products from developing countries in 2011 (β = 1.91, − 12.14 to 8.33) and in 2015 (β = 0.63, − 11.13 to 12.38). Association between higher GDP per capita and willingness to pay more was statistically significant in 2011 (0.81 pp., 0.49 to 1.13) and in 2015 (0.90 pp., 0.56 to 1.24).

Associations between the independent variables and percentage change of all three Development Views from 2011 to 2015 were non-significant (Table 4). For a sensitivity analysis with alternative classification of Development Views, see Additional file 1: Table S4.

**Table 4:** Association of percentage change in Development Views with migration status and percentage change in GDP per capita in the EU, 2011 to 2015

	Percentage change in ‘Very important’		Percentage change in ‘Increase beyond promise’		Percentage change in ‘Pay more’	
	β (95% CI)	P	β (95% CI)	P	β (95% CI)	P
Migration status						
Other EU country	(referent)					
Arrival country	21.00 (−6.00 to 48.00)	0.121	45.90 (−27.37 to 119.17)	0.208	7.80 (−10.17 to 25.77)	0.378
Destination country	−0.52 (− 25.52 to 24.49)	0.966	− 13.22 (− 81.08 to 54.64)	0.691	10.80 (−5.85 to 27.44)	0.193
Percentage change in GDP per capita, 2011 to 2015	0.38 (−0.79 to 1.54)	0.509	2.63 (−0.54 to 5.79)	0.099	0.57 (−0.20 to 1.35)	0.141

Shown for each covariate are regression coefficient β, 95% CI for β, and *p*-value of statistical significance

## Discussion

The present study investigated three aspects of EU citizens’ attitudes towards development aid. Across these issues in 2011 and 2015, support was generally highest in Scandinavia and Western Europe. Lowest percentages were recorded in some Eastern European nations, particularly Bulgaria and the Baltic States. In the EU27, from 2011 to 2015, support for all investigated issues increased. In 2015, the belief that development assistance is ‘very important’ was significantly higher in countries where migrants first arrived compared to other EU Member States, with a trend towards this association also apparent in 2011.

In describing strong support for helping people in developing countries (‘very important’), considerable differences in opinions were found between countries. This finding is consistent with previous reports of Eurobarometer survey results [15, 16]. The high levels of agreement in Sweden, for example, have been explained with reference to a collective national feeling or social norm regarding the importance of supporting the poor in developing countries, as well as widespread trust in governmental institutions spending aid effectively [31]. In contrast, stagnation of economic development in Latvia and Lithuania [32], may naturally elevate the importance of helping the poor domestically over assisting those living overseas.

A possible effect of migration was only apparent in arrival countries, where there was higher support for helping people in developing countries compared to other EU countries. This might be because arrival country citizens are more likely to see migrants in their worst physical states, due to the ordeals of their journeys [33]. When comparing Italy, which is an arrival country, to other EU countries, press coverage of migration was more often focused on humanitarian and migrant health themes [23]. These include journey-related injuries, particularly hypothermia-induced problems, as well as pregnancy related complications due to poor access to healthcare during the journey and upon arrival in EU communities [34]. Increased exposure of arrival country citizens to these issues may be a possible factor explaining higher levels of sympathy.

On the other hand, feelings towards immigration are generally known to be more negative in Southern Europe than Western Europe [35]. Therefore, arrival country citizens may have been more in favour of helping people in developing countries in the hope this might stave off the influx of migrants. Whether such thoughts are valid is debatable because of the complex relationship between development assistance and levels of migration [36]. Respondents’ views on the other measures used in the study (increasing promised levels of aid and spending more on products from developing countries) are bound to be even more complex, as they are more likely to be affected by respondents’ level of knowledge, as well as economic status and political outlooks.

The positive association between GDP per capita and citizens’ attitudes towards the importance of development aid becomes highly interesting in view of varying results reported in the literature. Several studies have agreed that income and support for development aid are positively associated in individual-level analyses [37, 38]. When controlling for individual-level factors though, Paxton & Knack determined that on the country-level, a US$1000 increase in GDP per capita of a nation decreased the probability of its citizens supporting development aid by 4% [38]. GDP per capita may be influenced extensively by other variables, which could not be controlled for in the present study. In any case, it appears natural to expect that greater proportions of people in richer countries, owing to purchasing power differentials, are willing to pay more for products from developing countries to help the people living there.

Some important limitations of the present study should be acknowledged. Using survey data implies risk for selection bias, and face-to-face interviews may evoke a response bias towards what is socially desirable [39, 40]. Furthermore, Eurobarometer surveys do not question respondents’ knowledge on purpose or amounts of aid [41, 42]. The public tends to overestimate levels of development assistance, with a third of UK citizens holding the belief that their government spent five to 10 % of its GNI on aid in 2011; hence it is difficult to gauge how this tendency, as well as the public’s perceptions and knowledge regarding different types of aid may have impacted results [43]. Studies of citizens’ perceptions of national level phenomena can be influenced by factors that are external or indirectly related to people’s experiences of the phenomenon under study, such as media representation, nature of political discourse, and the national mood in general. For example, the national mood in different countries may still be suffering due to residual sentiments, and media representations of these, around the financial crisis; however, these complex dynamics are difficult to capture in such a survey [44, 45]. Methodologically, care was taken to choose arrival countries based on trends reported by Frontex, and destination countries as per asylum application statistics provided by Eurostat [27]; other possible methods of designating migration status might have given different results.

The present study draws its main strengths from the consistency of data collection methods across countries and years. Apart from the Eurobarometer survey results, all other data used for the analysis were taken from Eurostat, implying that methods of obtaining this data in 2011 and 2015 were similar. Additionally, the timing of the surveys was suitable; wave 76.1 in September 2011 reflected attitudes prior to major global increases in forced displacement, and wave 84.4 from December 2015 captured opinions just months after breaking news coverage of the European migrant crisis.

## Conclusions

Although relationships between recent migratory trends and opinions on development aid are not straightforward, policy makers should be encouraged to continue to tie-in advocacy for development assistance with migration policies. Careful framing and presentation of such policies might also improve peoples’ understanding of how development aid and migration relate. The general increase in public support for development aid from 2011 to 2015 should provide impetus for EU institutions and Member State policy makers to pursue aid targets towards the 0.7% ODA per GNI mark. At a time of rising popularity of right-wing nationalism in some EU countries, this could help emphasise core European values such as equity and solidarity, which also form the very foundations of global accords such as the Sustainable Development Goals [46]. The consequent natural net increase in ODA-H spending would be critical for the pursuit of the major Global Health targets in the coming decades.

## Additional file

Additional file 1: Supplementary Tables. (DOCX 31 kb)

## Abbreviations

DAC: Development Assistance Committee
EU: European Union
EU27: The 27 Member States of the European Union
GDP: Gross domestic product
GNI: Gross national income
ODA: Official development assistance
ODA-H: Official development assistance for health
OECD: Organization for economic co-operation and development
