# Supplementary material for: European Union citizens’ views on development assistance for developing countries, during the recent migrant crisis in Europe
Source: Global Health. 2018 Jun 28;14:61. doi: 10.1186/s12992-018-0378-1 (PMC6022484; doi:10.1186/s12992-018-0378-1)
Supplement: Supplementary file 1 — Supplementary Tables. (DOCX 31 kb) [file 12992_2018_378_MOESM1_ESM.docx]

**SUPPLEMENTARY TABLES**

| Supplementary Table 1. Percentage of each country’s population of opinion that helping people in developing countries is ‘very important’ or ‘fairly important’, and that the EU should go beyond or keep its promise to increase development aid, 2011 and 2015. | | | | | | |
| --- | --- | --- | --- | --- | --- | --- |
| Country | **Opinion ‘very & fairly important’, 2011**  **(%, 95% CI)** | **Opinion ‘very & fairly important’, 2015**  **(%, 95% CI)** | **Change in ‘very & fairly important’, 2011-2015 (%)** | **Opinion ‘increase beyond & keep promise’, 2011**  **(%, 95% CI)** | **Opinion ‘increase beyond & keep promise’, 2015**  **(%, 95% CI)** | **Change in ‘increase beyond & keep promise’, 2011-2015 (%)** |
| Austria (AT) | 84.4 (81.9 - 86.7) | 86.4 (84.0 - 88.5) | 2.3 | 77.0 (74.2 - 79.6) | 66.2 (63.0 - 69.3) | -14.0 |
| Belgium (BE) | 83.0 (80.5 - 85.3) | 85.2 (82.8 - 87.4) | 2.6 | 60.7 (57.6 - 63.8) | 65.4 (62.3 - 68.5) | 7.8 |
| Bulgaria (BG) | 75.1 (72.2 - 77.7) | 72.5 (69.6 - 75.2) | -3.4 | 32.9 (30.0 - 36.0) | 25.6 (23.0 - 28.4) | -22.3 |
| Cyprus (CY) | 95.0 (92.7 - 96.6) | 90.8 (87.6 - 93.2) | -4.5 | 47.2 (42.9 - 51.6) | 69.2 (64.5 - 73.6) | 46.6 |
| Czech Republic (CZ) | 82.6 (80.1 - 84.8) | 78.5 (75.7 - 81.0) | -5.0 | 59.9 (56.8 - 62.9) | 54.0 (50.8 - 57.3) | -9.8 |
| Denmark (DK) | 90.2 (88.1 - 92.1) | 92.2 (90.2 - 93.8) | 2.1 | 74.0 (70.9 - 76.8) | 74.6 (71.5 - 77.5) | 0.9 |
| Estonia (EE) | 73.8 (71.0 - 76.5) | 75.7 (72.8 - 78.3) | 2.5 | 53.6 (50.4 - 56.8) | 53.5 (50.2 - 56.8) | -0.2 |
| Finland (FI) | 90.8 (88.7 - 92.6) | 91.1 (89.1 - 92.8) | 0.4 | 70.6 (67.4 - 73.7) | 60.7 (57.4 - 64.0) | -14.0 |
| France (FR) | 81.9 (79.4 - 84.2) | 86.1 (83.8 - 88.1) | 5.1 | 69.5 (66.6 - 72.2) | 76.4 (73.6 - 79.0) | 10.0 |
| Germany (DE) | 92.0 (90.4 - 93.4) | 92.8 (91.1 - 94.2) | 0.8 | 67.0 (64.2 - 69.7) | 74.4 (71.8 - 76.9) | 11.1 |
| Greece (EL) | 82.9 (80.3 - 85.2) | 84.4 (81.9 - 86.6) | 1.8 | 60.2 (57.1 - 63.3) | 53.4 (50.0 - 56.7) | -11.4 |
| Hungary (HU) | 74.5 (71.6 - 77.2) | 73.3 (70.3 - 76.1) | -1.6 | 49.4 (46.1 - 52.6) | 62.0 (58.8 - 65.1) | 25.6 |
| Ireland (IE) | 85.2 (82.8 - 87.3) | 91.2 (89.2 - 92.9) | 7.1 | 49.4 (46.2 - 52.5) | 75.8 (72.9 - 78.4) | 53.4 |
| Italy (IT) | 84.1 (81.6 - 86.2) | 89.9 (87.5 - 91.8) | 6.9 | 63.9 (60.9 - 66.8) | 66.4 (63.1 - 69.5) | 3.9 |
| Latvia (LV) | 81.2 (78.6 - 83.4) | 66.4 (63.0 - 69.5) | -18.2 | 63.5 (60.5 - 66.4) | 56.3 (52.9 - 59.7) | -11.3 |
| Lithuania (LT) | 79.2 (76.6 - 81.6) | 73.8 (70.6 - 76.7) | -6.9 | 50.0 (46.9 - 53.1) | 46.2 (42.6 - 49.8) | -7.6 |
| Luxembourg (LU) | 92.2 (89.3 - 94.3) | 95.8 (93.5 - 97.3) | 3.9 | 80.3 (76.3 - 83.8) | 83.9 (79.7 - 87.4) | 4.5 |
| Malta (MT) | 87.9 (84.3 - 90.8) | 92.2 (89.0 - 94.6) | 4.9 | 52.4 (47.5 - 57.3) | 78.2 (74.0 - 81.9) | 49.1 |
| Netherlands (NL) | 86.9 (83.9 - 89.5) | 92.2 (90.3 - 93.7) | 6.1 | 60.4 (56.4 - 64.2) | 72.4 (69.3 - 75.3) | 20.0 |
| Poland (PL) | 91.9 (90.1 - 93.4) | 86.7 (84.4 - 88.8) | -5.6 | 65.4 (62.3 - 68.4) | 56.8 (53.6 - 59.9) | -13.2 |
| Portugal (PT) | 87.6 (85.4 - 89.5) | 93.5 (91.8 - 94.9) | 6.7 | 58.9 (55.9 - 61.9) | 65.6 (62.5 - 68.5) | 11.2 |
| Romania (RO) | 76.9 (74.1 - 79.4) | 88.2 (85.9 - 90.1) | 14.7 | 45.9 (42.9 - 49.0) | 70.8 (67.8 - 73.7) | 54.2 |
| Slovakia (SK) | 84.3 (81.7 - 86.6) | 78.8 (76.1 - 81.4) | -6.5 | 63.3 (60.0 - 66.5) | 56.4 (53.2 - 59.6) | -10.9 |
| Slovenia (SI) | 70.8 (67.8 - 73.6) | 80.0 (77.2 - 82.5) | 13.0 | 57.2 (54.1 - 60.3) | 67.1 (63.9 - 70.1) | 17.3 |
| Spain (ES) | 87.7 (85.5 - 89.6) | 93.7 (91.7 - 95.2) | 6.9 | 54.1 (51.0 - 57.2) | 72.5 (69.2 - 75.6) | 33.9 |
| Sweden (SE) | 96.6 (95.1 - 97.6) | 98.2 (96.6 - 99.0) | 1.6 | 79.6 (76.6 - 82.3) | 78.8 (74.9 - 82.1) | -1.0 |
| United Kingdom (UK) | 80.7 (78.2 - 83.1) | 85.8 (83.5 - 87.8) | 6.3 | 54.8 (51.7 - 57.9) | 68.7 (65.8 - 71.5) | 25.3 |
| EU27 | **85.3 (84.7 - 86)** | **88.2 (87.6 - 88.8)** | **3.4** | **61.4 (60.5 - 62.4)** | **68.2 (67.3 - 69.1)** | **11.0** |
|  | | | |  |  |  |

| Supplementary Table 2. Percentage of each country’s population prepared to pay more for products from developing countries to help the people living there, 2011 and 2015. | | | |
| --- | --- | --- | --- |
| Country | **Opinion ‘pay more’, 2011**  **(%, 95% CI)** | **Opinion ‘pay more’, 2015**  **(%, 95% CI)** | **Change 2011-2015 (%)** |
| Austria (AT) | 53.5 (50.3 - 56.6) | 55.4 (52.0 - 58.8) | 3.6 |
| Belgium (BE) | 54.3 (51.1 - 57.5) | 58.1 (54.9 - 61.3) | 7.0 |
| Bulgaria (BG) | 22.2 (19.6 - 25.1) | 15.5 (13.4 - 17.8) | -30.3 |
| Cyprus (CY) | 48.3 (43.9 - 52.6) | 51.9 (47.2 - 56.7) | 7.6 |
| Czech Republic (CZ) | 37.3 (34.4 - 40.4) | 34.1 (31.0 - 37.3) | -8.7 |
| Denmark (DK) | 65.7 (62.4 - 68.8) | 69.0 (65.8 - 72.0) | 5.0 |
| Estonia (EE) | 31.4 (28.5 - 34.5) | 36.3 (33.1 - 39.6) | 15.6 |
| Finland (FI) | 69.4 (66.2 - 72.4) | 69.6 (66.4 - 72.6) | 0.3 |
| France (FR) | 51.7 (48.6 - 54.9) | 53.7 (50.5 - 56.9) | 3.8 |
| Germany (DE) | 63.3 (60.5 - 66.1) | 69.8 (67.0 - 72.5) | 10.2 |
| Greece (EL) | 44.5 (41.4 - 47.7) | 33.0 (30.0 - 36.3) | -25.8 |
| Hungary (HU) | 27.5 (24.7 - 30.5) | 24.7 (22.0 - 27.7) | -10.1 |
| Ireland (IE) | 39.7 (36.6 - 42.8) | 49.2 (46.0 - 52.5) | 24.1 |
| Italy (IT) | 37.5 (34.6 - 40.6) | 35.7 (32.5 - 39.1) | -4.8 |
| Latvia (LV) | 28.0 (25.3 - 30.9) | 26.0 (23.1 - 29.1) | -7.4 |
| Lithuania (LT) | 32.0 (29.2 - 34.9) | 22.7 (19.8 - 25.9) | -29.1 |
| Luxembourg (LU) | 74.3 (70.0 - 78.1) | 80.0 (75.5 - 83.8) | 7.6 |
| Malta (MT) | 37.3 (32.7 - 42.1) | 45.4 (40.6 - 50.2) | 21.7 |
| Netherlands (NL) | 79.4 (75.9 - 82.5) | 77.0 (74.0 - 79.7) | -3.0 |
| Poland (PL) | 34.8 (31.7 - 37.9) | 24.8 (22.1 - 27.7) | -28.7 |
| Portugal (PT) | 20.4 (18.0 - 23) | 17.8 (15.5 - 20.4) | -12.8 |
| Romania (RO) | 19.4 (17.0 - 21.9) | 26.7 (23.9 - 29.7) | 38.0 |
| Slovakia (SK) | 33.9 (30.8 - 37.2) | 28.7 (25.9 - 31.8) | -15.3 |
| Slovenia (SI) | 38.5 (35.5 - 41.6) | 33.5 (30.4 - 36.7) | -12.9 |
| Spain (ES) | 41.4 (38.3 - 44.5) | 45.2 (41.7 - 48.7) | 9.1 |
| Sweden (SE) | 76.4 (73.4 - 79.2) | 80.3 (76.5 - 83.6) | 5.1 |
| United Kingdom (UK) | 53.1 (50.0 - 56.2) | 63.6 (60.6 - 66.6) | 19.9 |
| EU27 | **47.3 (46.3 - 48.2)** | **49.5 (48.5 - 50.5)** | **4.7** |
| All percentages and confidence intervals are given to one decimal place. | | | |

| **Supplementary Table** **3.** Association of Development Views with migration status and GDP per capita in the EU, 2011 & 2015. | | | | | | | | |
| --- | --- | --- | --- | --- | --- | --- | --- | --- |
|  | **Very & fairly important 2011** | | **Very & fairly important**  **2015** | | **Increase beyond & keep promise**  **2011** | | **Increase beyond & keep promise**  **2015** | |
|  | β  (95% CI) | p | β  (95% CI) | p | β  (95% CI) | p | β  (95% CI) | p |
| *Migration status* |  |  |  |  |  |  |  |  |
| Other EU country | (referent) |  |  |  |  |  |  |  |
| Arrival country | 2.04  (-3.89 to 7.95) | 0.484 | 5.23  (-1.38 to 11.84) | 0.116 | -6.62  (-14.55 to 1.30) | 0.097 | 0.48  (-9.59 to 10.55) | 0.922 |
| Destination country | 0.79  (-5.57 to 7.15) | 0.800 | -0.34  (-7.18 to 6.49) | 0.919 | 5.04  (-3.48 to 13.56) | 0.234 | -1.67  (-12.08 to 8.74) | 0.743 |
| GDP/ capita (per 1,000 EUR) | 0.24  (0.06 to 0.43) | 0.012 | 0.36  (0.17 to 0.56) | 0.001 | 0.34  (0.09 to 0.58) | 0.010 | 0.52  (0.23 to 0.81) | 0.001 |
| Shown for each covariate are regression coefficient β, 95% CI for β, and p value of statistical significance. β coefficients are adjusted for all variables shown in the table. | | | | | | | | |

| **Supplementary Table** **4.** Association of percentage change in Development Views with migration status and percentage change in GDP per capita in the EU, 2011 to 2015. | | | | |
| --- | --- | --- | --- | --- |
|  | **Change in**  **‘Very & Fairly important’** | | **Change in**  **‘Increase beyond & Keep promise’** | |
|  | β (95% CI) | p | β (95% CI) | p |
| *Migration status* |  |  |  |  |
| Other EU country | (referent) |  |  |  |
| Arrival country | 2.52  (-4.96 to 10.00) | 0.493 | 15.75  (-6.76 to 38.27) | 0.161 |
| Destination country | 0.64  (-6.29 to 7.56) | 0.85 | -0.97  (-21.82 to 19.89) | 0.925 |
| GDP/ capita (thousands) | -0.003  (-0.33 to 0.32) | 0.986 | 0.85  (-0.12 to 1.83) | 0.082 |
| Shown for each covariate are regression coefficient β, 95% CI for β, and p value of statistical significance. | | | | |
